# Antimicrobial Resistance and Molecular Epidemiology of ESBL-Producing *Escherichia coli* Isolated from Outpatients in Town Hospitals of Shandong Province, China

**DOI:** 10.3389/fmicb.2017.00063

**Published:** 2017-01-24

**Authors:** Zengmin Miao, Song Li, Lei Wang, Wengang Song, Yufa Zhou

**Affiliations:** ^1^Department of Life Sciences, Taishan Medical University Tai'an, China; ^2^Department of Basic Medicine, Taishan Medical University Tai'an, China; ^3^Department of Pediatrics, Maternal and Child Health hospital of Laiwu Laiwu, China; ^4^Disease Controlling Center, Veterinary Bureau of Daiyue Tai'an, China

**Keywords:** outpatient, antimicrobial resistance, ESBL, ST, town hospital

## Abstract

This study aimed to investigate antimicrobial resistance and molecular epidemiology of extended-spectrum β-lactamase (ESBL)-producing *Escherichia coli* (*E. coli*) isolated from outpatients in town hospitals of Shandong province, China. Antimicrobial susceptibility of ESBL-producing *E. coli* was tested using the disk diffusion and resistance genes encoding for β-lactamases (*bla*_TEM_, *bla*_CTXM_, and *bla*_SHV_) were detected by polymerase chain reaction (PCR). Multilocus sequence typing (ST) of ESBL-producing *E. coli* was analyzed in this study. Our results showed that of 320 *E. coli* isolates, 201 carried ESBL genes (201/320, 62.8%), and these isolates all carried *bla*_CTX-M_ genes, the most common being *bla*_CTX-M-14_ (116/201, 57.7%), followed by *bla*_CTX-M-55_ (47/201, 23.4%) and *bla*_CTX-M-15_ (31/201, 15.4%). ESBL-producing *E. coli* exhibited highly resistant to penicillin derivatives, fluoroquinolones, folate pathway inhibitors, and third-generation cephalosporins, but no carbapenem-resistant isolates were found in this study. Forty-two STs were found among the 201 ESBL-producing *E. coli*, and the most common ST was ST131 (27/201, 13.4%), followed by ST405 (19/201, 9.5%) and ST69 (15/201, 7.5%). Taken together, a high isolation rate of ESBL-producing *E. coli* (62.8%) was found among outpatients in town hospitals. *bla*_CTX-M_ gene was most dominant and was composed of a variety of subtypes. No dominant ST was detected among ESBL-producing *E. coli*, indicating that these ESBL-producing *E. coli* isolates derive from different clones.

## Introduction

Beta-lactam antimicrobials are first line anti-bacterial infection drugs for humans due to their high potency, broad anti-bacterial spectrum, and minimal side effects. They are widely used in the treatment of various infections, such as those of the lungs, urinary tract, and the bloodstream. However, widespread use of antibiotics has intensified the problem of antibiotic resistance in bacteria (Paterson and Bonomo, 2005; Biedenbach et al., 2014; D'Angelo et al., 2016). The production of extended-spectrum beta-lactamases (ESBLs) is an important mechanism of antimicrobial resistance in Enterobacteriaceae, especially *Escherichia coli* (*E. coli*) and *Klebsiella pneumoniae* (*K. pneumoniae*), and the enzyme can hydrolyze penicillin, cephalosporin, and monocyclic amide antibiotics, but its activity is usually inhibited by beta-lactamase inhibitors, such as sulbactam, clavulanic acid, and tazobactam (Bush et al., 1995; Bradford, 2001).

Currently, over hundreds of ESBLs have been identified; the most prevalent genotypes are *bla*_TEM_, *bla*_SHV_, and *bla*_CTX−M_. Within the past decade, the genotype *bla*_CTX−M_ has rapidly increased and is now widely found in clinically isolated *E. coli* across the world (Paterson and Bonomo, 2005; Livermore et al., 2007). In practice, *bla*_CTX−M_ genes have already become the major ESBL genotype in American, European, and Asian countries (Pitout et al., 2005; Livermore et al., 2007; Ben-Ami et al., 2009; Zhang et al., 2014). Emergence of community-associated infections caused by ESBL-producing *E. coli* has been reported in Europe and the United States (Ben-Ami et al., 2009). Moreover, relevant studies from Oceania, Asia, and South America have also reported that ESBL-positive *E. coli* are the key pathogens in community-onset infections (Baas and Ahmad, 2001; Bell et al., 2002; Munday et al., 2004; da Silva Dias et al., 2008; Baurin et al., 2009; Rawat et al., 2013).

Numerous studies in China have already demonstrated that ESBL-producing *E. coli* in tertiary and county hospitals is becoming an epidemic (Xiao et al., 2011, 2012, 2013; Zhang et al., 2014; Liu et al., 2015). Previous studies that monitored infections in tertiary hospitals of China indicated that the prevalence of ESBL-producing *E. coli* was rapidly on the rise, increasing from an ESBL-positive rate of <20% in 2000 to 72.2% in 2011 (Xiao et al., 2011, 2012, 2013). A similar study that examined infections in county hospitals across China also reported an ESBL-positive rate of up to 46.5% in *E. coli* (Zhang et al., 2014). However, these studies were focused on city hospitals, and there are very few reports that have examined ESBL-producing *E. coli* in town hospitals of rural areas in China. Therefore, this study was undertaken to investigate drug-resistance and molecular epidemiology of ESBL-producing *E. coli* isolated from outpatients in town hospitals of Shandong province, in order to provide comprehensive and reliable epidemiological information for preventing dissemination of resistance genes.

## Materials and methods

### Ethics statement

This study was in compliance with the various requirements of the Research Ethics Committee of Taishan Medical University (Permit No.: TSMC20141012). All participants signed an informed consent.

### Sample collection

**Figure 1:**
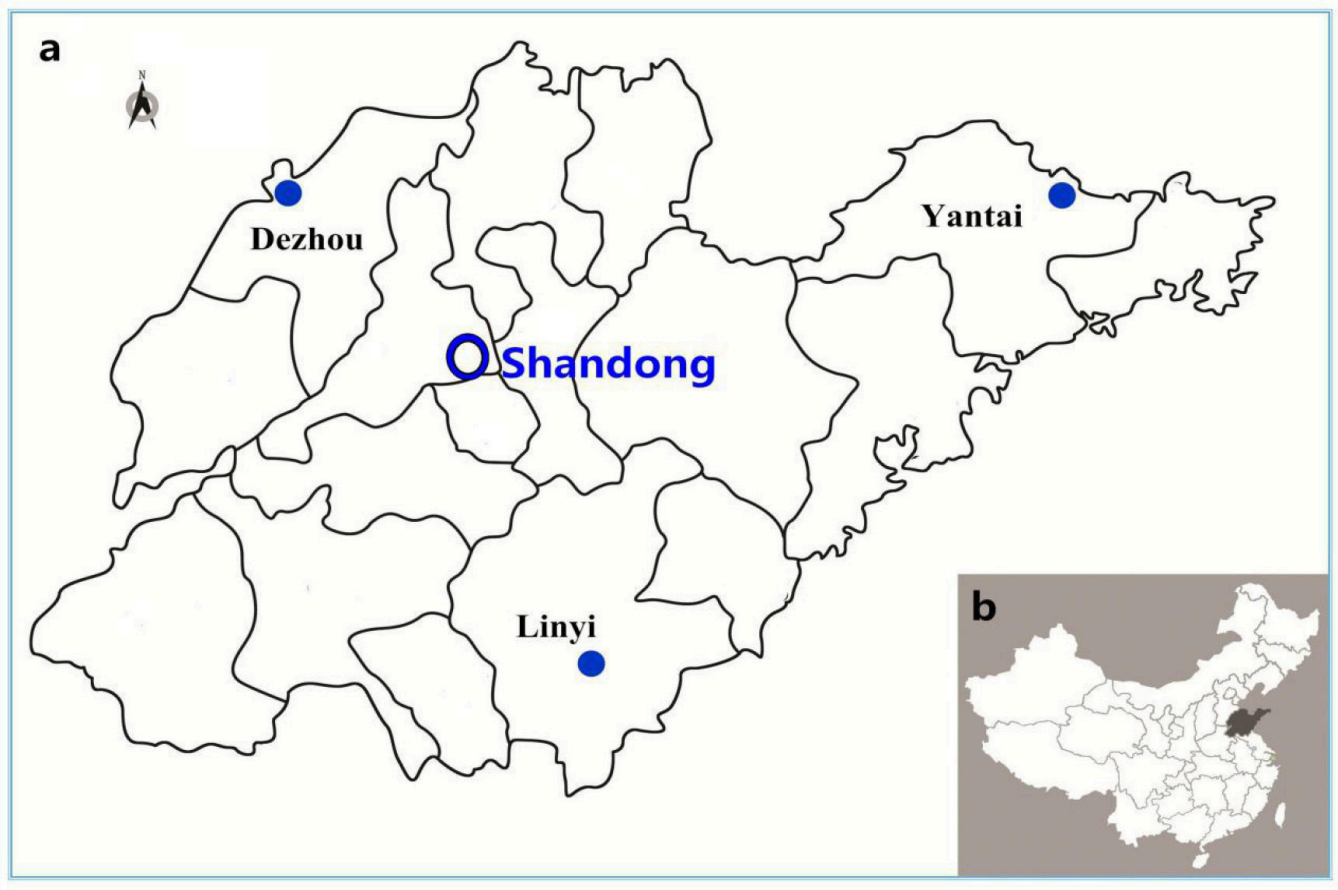
**Sampling sites in this study. (A):** The enlarged map of Shandong province, in which sampling sites in three administrative districts was marked. **(B)**: The location of Shandong province was highlighted in China.

Sputum and urine samples of outpatients were collected from 15 town hospitals across three regions of the Shandong province (five hospitals per region from October 2014 to September 2015), for *E. coli* isolation (Figure 1). The outpatients were selected according to the following three conditions: (1) they had not stayed at the hospital within the past 3 months, (2) they had no long-term intubation, and (3) they had not taken antimicrobial medication for over 72 h before treatment.

### Isolation and identification of *E. coli*

Samples were transported back to the lab on ice within 6–10 h of collection, for *E. coli* isolation and identification. Samples were inoculated onto MacConkey agar plates using sterile cotton swabs and were incubated overnight at 37°C in aerobic conditions. Five single red colonies from each patient sample were selected for further colony purification, and the colonies were subsequently identified using conventional biochemical methods and API20 assays (bioMérieux, Durham, NC, USA). All positively identified *E. coli* strains (one strain per patient) were stored at −80°C in Luria-Bertani (LB) broth containing 30% glycerol.

### Antimicrobial susceptibility and ESBL phenotypic confirmatory tests

*E. coli* susceptibility to 17 antibiotics, including ampicillin, piperacillin, ampicillin-sulbactam, piperacillin-tazobactam, cefotaxime, cefriaxone, cefuroxime, cefepime, ceftazidime, aztreonam, imipenem, meropenem, amikacin, gentamicin, ciprofloxacin, levofloxacin, and trimethoprim-sulfamethoxazole, was tested using disk diffusion. All drug susceptibility testing were performed in accordance with the CLSI 2014 criteria (Clinical Laboratory Standards Institute, 2014). *E. coli* ATCC25922 and *K. pneumoniae* ATCC700603 were used as quality control strains.

ESBL phenotypic confirmatory test was performed on *E. coli* using the double-disc synergy procedure with paper disks that contained ceftazidime and cefotaxime alone, or in combination with clavulanic acid (30 μg ceftazidime, 30/10 μg ceftazidime/clavulanic acid, 30 μg cefotaxime, 30/10 μg cefotaxime/clavulanic acid) (Oxoid Limited, UK; Clinical Laboratory Standards Institute, 2014).

### Bacterial DNA extraction

Single colonies of ESBL-producing *E. coli* were inoculated into LB media and cultured overnight at 37°C with 220 rpm shaking. Bacterial culture (1 mL) was transferred to an Eppendorf tube, centrifuged at 12,000 rpm for 5 min, before the pellet was resuspended in 60 μl of sterile ultrapure water. The solution was then placed in boiling water for 10 min, immediately transferred to an ice bath for 5 min, and centrifuged at 12,000 rpm for 5 min to obtain the extracted bacterial DNA in the supernatant.

### Detection of beta-lactamase gene by PCR

Polymerase chain reaction (PCR) amplification for the beta-lactamase genes (TEM, SHV, and CTX-M) were carried out as previously described (Yu et al., 2007; Dallenne et al., 2010; Sun et al., 2010; Zhang et al., 2011, 2014). The PCR products were sequenced following purification, and ESBL genotype was determined by comparing to GenBank sequences (http://www.ncbi.nlm.nih.gov/BLAST).

### MLST

Multilocus sequence typing of the ESBL-producing *E. coli* was performed according to the experimental procedures on the Environmental Research Institute, University College Cork website (http://mlst.ucc.ie/mlst/dbs/Ecoli; Lau et al., 2008a). The *E. coli* strains were grouped according to the eBURST algorithm based on their allelic properties, where strains with the same six out of seven alleles were assigned to the same group (Feil et al., 2004).

### Statistical analysis

Statistical analysis was performed using SAS 8.2 (SAS Institute, Cary, NC, USA). Continuous variables and categorical variables were compared using the Student's *t*-test and chi-squared test or Fisher's exact test, respectively. A two-tailed *P* < 0.05 was considered to be statistically significant.

## Results

### Outpatient demographics

A total of 320 outpatients were recruited in this study, including 110 from Yantai (YT), 90 from Dezhou (DZ), and 120 from Linyi (LY). Among 320 outpatients aged 10–85 years, there were 32 between 10 and 18 years of age (10.0%), 161 between 19 and 45 years (50.3%), 107 between 46 and 65 years (33.4%), and 20 over 65 years (6.3%). There were more male (185/320, 57.8%) than female (135/320, 42.2%) outpatients.

### Isolation and identification of *E. coli*

A total of 320 *E. coli* isolates were recovered, comprising 231 isolated from urine (72.2%) and 89 (27.8%) from sputum. Among these 320 *E. coli*, 201 carried ESBL genes (201/320, 62.8%), including 67 (67/110, 60.9%), 79 (79/120, 65.8%), and 55(55/90, 61.1%) isolates from YT, LY, and DZ, respectively. The isolation rates of ESBL-producing *E. coli* among three regions did not differ significantly (*P* > 0.05), but the isolation rate of ESBL-producing *E. coli* in urine (170/231, 73.6%) was significantly higher than that in sputum (31/89, 34.8%; *P* < 0.05).

### Antimicrobial resistance characteristics

All of the 320 *E. coli* were susceptible to imipenem and meropenem, and showed high resistance rates to ampicillin (269/320, 84.1%), piperacillin (251/320, 78.4%), ciprofloxacin (238/320, 74.4%), levofloxacin (236/320, 73.8%), trimethoprim-sulfamethoxazole (230/320, 71.9%), gentamicin (222/320, 69.4%), cefotaxime (216/320, 67.5%), ceftriaxone (209/320, 65.3%), and cefuroxime (206/320, 64.4%). By contrast, these isolates exhibited low resistance rates to piperacillin/tazobactam (8/320, 2.5%), amikacin (8/320, 2.5%), ceftazidime (76/320, 23.8%), and cefepime (78/320, 24.4%). In addition, ESBL-producing *E. coli* showed significantly higher resistance rates to most antibiotics compared with those of non-ESBL-producing *E. coli* (*P* < 0.0001). However, the low resistance rate against piperacillin-tazobactam and high resistance rate to gentamicin in the two populations did not differ significantly (*P* > 0.05; Table 1).

**Table 1:** **Rates of antimicrobial resistance among ***E. coli*** isolates**.

**Antimicrobial agents**		**No. of isolates (%)**			***P***
		**Total (*n* = 320)**	**ESBL (*n* = 201)**	**Non-ESBL (*n* = 119)**	
Penicillin derivatives	AMP	269 (84.1)	201 (100.0)	68 (57.1)	<0.0001
	PRL	251 (78.4)	201 (100.0)	50 (42.0)	<0.0001
β-Lactam/β-lactamase inhititor combinations	SAM	126 (39.4)	112 (55.7)	14 (11.8)	<0.0001
	TZP	8 (2.5)	5 (2.5)	3 (2.5)	–
Cephalosporins	CRO	209 (65.3)	192 (95.5)	17 (14.3)	<0.0001
	CXM	206 (64.4)	195 (97.0)	11 (9.2)	<0.0001
	CEF	78 (24.4)	75 (37.3)	3 (2.5)	<0.0001
	CTX	216 (67.5)	198 (98.5)	18 (15.1)	<0.0001
	CAZ	76 (23.8)	72 (35.8)	4 (3.4)	<0.0001
Monobactams	ATM	98 (30.6)	95 (47.3)	3 (2.5)	<0.0001
Carbapenems	IPM	0 (0)	0 (0)	0 (0)	–
	MEM	0 (0)	0 (0)	0 (0)	–
Amimoglycosides	AK	8 (2.5)	8 (4.0)	0 (0)	<0.0001
	GM	222 (69.4)	141 (70.1)	81 (68.1)	>0.05
Fluoroquinolones	CIP	238 (74.4)	192 (95.5)	46 (38.7)	<0.0001
	LEV	236 (73.8)	191 (95.0)	45 (37.8)	<0.0001
Folate pathway inhibitors	SXT	230 (71.9)	190 (94.5)	40 (33.6)	<0.0001

*AMP, ampicillin; PRL, piperacillin; SAM, ampicillin-sulbactam; TZP, piperacillin-tazobactam; CTX, cefotaxime; CRO, cefriaxone; CXM, cefuroxime; CEF, cefepime; CAZ, ceftazidime; ATM, aztreonam; IPM, imipenem; MEM, meropenem; AK, amikacin; GM, gentamicin; CIP, ciprofloxacin; LEV, levofloxacin; SXT, trimethoprim-sulfamethoxazole*.

### ESBL gene characteristics

All of the 201 ESBL-producing *E. coli* strains carried *bla*_CTX-M_ genes, with the most common being *bla*_CTX-M-14_ (116/201, 57.7%), followed by *bla*_CTX-M-55_ (47/201, 23.4%) and *bla*_CTX-M-15_ (31/201, 15.4%). Additionally, 122 of the 201 ESBL-producing *E. coli* strains simultaneously carried *bla*_TEM-1_ genes. *bla*_SHV_ genes were not detected in this study. There was no significant difference in the prevalence of *bla*_CTX-M_ genes among the ESBL-producing *E. coli* isolated from the three regions (*P* > 0.05; Table 2).

**Table 2:** **ESBL genes in 201 ESBL-positive ***E. coli*** isolates from three administrative districts**.

**Types of ESBL**		**No. of isolates (%)**				***P***
		**Total**	**YT**	**DE**	**LY**	
ESBL		201 (100.0)	67 (100.0)	55 (100.0)	79 (100.0)	–
CTX-M		201 (100.0)	67 (100.0)	55 (100.0)	79 (100.0)	–
	CTX-M-14	116 (57.7)	40 (59.7)	28 (50.9)	48 (60.8)	>0.05
	CTX-M-55	47 (23.4)	15 (22.4)	12 (21.8)	20 (25.3)	>0.05
	CTX-M-15	31 (15.4)	10 (14.9)	8 (14.5)	13 (16.5)	>0.05
	CTX-M-1	1 (0.5)	0 (0.0)	0 (0.0)	1 (1.3)	–
	CTX-M-3	1 (0.5)	0 (0.0)	0 (0.0)	1 (1.3)	–
	CTX-M-24	1 (0.5)	1 (1.3)	0 (0.0)	0 (0.0)	–
	CTX-M-27	1 (0.5)	1 (1.3)	0 (0.0)	0 (0.0)	–
	CTX-M-65	1 (0.5)	0 (0.0)1 (1.3)	1 (1.3)	0 (0.0)	–
	CTX-M-79	1 (0.5)	0 (0.0)	0 (0.0)	1 (1.3)	–
	CTX-M-101	1 (0.5)	0 (0.0)	0 (0.0)	1 (1.3)	–

*YT, Yantai; DE, Dezhou; LY, Linyi*.

### Multilocus sequence typing of ESBL-producing *E. coli*

**Figure 2:**
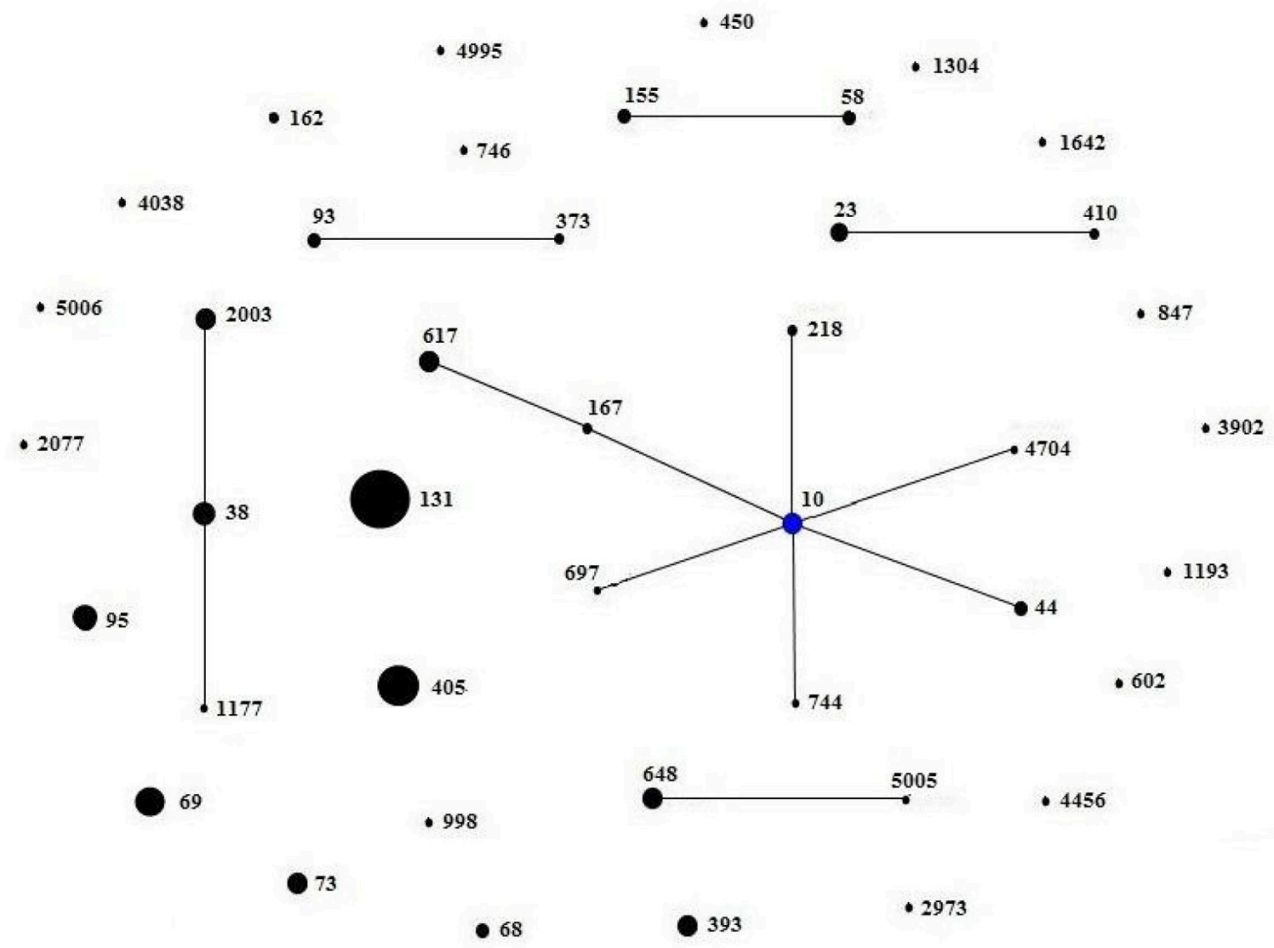
**Minimum spanning tree constructed based on the MLST profiles of ESBL-producing ***E. coli*****. There were 23 singletons, 5 groups (group1: ST58, ST155; group2: ST93, ST373; group3: ST23, ST410; group4: ST2003, ST38, ST1177; group5: ST648, ST5005), and 1 clonal complex (ST617, ST167, ST10, ST44, ST218, ST744, ST697, ST4704), which was radial. The blue dot in it indicated putative founder. The area of each black circle corresponded to the prevalence of the ST in the MLST data of this study.

A total of 42 different STs were found, which were grouped in 5 non-overlapping groups, 1 clonal complex, and 23 singletons (Figure 2). The most common ST were ST131 (27/201, 13.4%), followed by ST405 (19/201, 9.5%) and ST69 (15/201, 7.5%). There were 27 ST131 detected from town hospitals across the three regions, including 17 strains that were *bla*_CTX-M-14_-positive, 4 strains that were *bla*_CTX-M-15_-positive, 5 strains that were *bla*_CTX-M-55_-positive, and 1 strain that carried *bla*_CTX-M-3_ gene (Table 3).

**Table 3:** **Genotypes in MLST of 201 ESBL-producing ***E.coli*** isolates in this study**.

**ST**	**Total number**	**ESBL genes (number)**			
		**CTX-M-14**	**CTX-M-15**	**CTX-M-55**	**Others**
ST131	27	17	4	5	CTX-M-3 (1)
ST405	19	15	2	2	
ST69	15	9	3	3	
ST95	12	8	1	3	
ST38	11	8	1	2	
ST648	10	7	2	1	
ST617	10	7	1	2	
ST10	10	4	3	2	CTX-M-1 (1)
ST393	10	8	1	1	
ST73	10	6	2	2	
ST2003	10	6	2	2	
ST23	7	1	2	4	
ST44	4	2	1	1	
ST58	4		1	2	CTX-M-24 (1)
ST68	4	1	1	2	
ST93	4	1	2	1	
ST155	4	1	1	2	
ST162	2	1		1	
ST167	2	1		1	
ST218	2			1	
ST373	2	2			
ST410	2		1	1	
ST450	1	2			
ST602	1	1			
ST697	1			1	
ST744	1	1			
ST746	1				CTX-M-27 (1)
ST847	1			1	
ST998	1	1			
ST1177	1			1	
ST1193	1	1			
ST1304	1				CTX-M-79 (1)
ST1642	1	1			
ST2077	1			1	
ST2973	1	1			
ST3902	1				CTX-M-65 (1)
ST4038	1	1			
ST4456	1			1	
ST4704	1			1	
ST4995	1	1			
ST5005	1	1			
ST5006	1				CTX-M-101 (1)
	201	116	31	47	7

## Discussion

To our best knowledge, this study was the first time to investigate drug resistance and molecular characteristics of ESBL-producing *E. coli* from outpatients in town hospitals of Shandong province, China. The isolation rate of ESBL-producing *E. coli* in our study was 62.8%, which was similar with Zhao's (62.5%) and Wang's (67.8%) results conducted in Shanghai, China (Zhao et al., 2015; Wang et al., 2016), but higher than those reported in Argentina (18.1%), Mexico (48.4%), Chile (23.8%), and Brazil (12.8%; Gales et al., 2012). All ESBL-producing *E. coli* from these three regions carried *bla*_CTX-M_ genes, which was composed of 10 genotypes including *bla*_CTX-M-1, -3, -14, -15, -24, -27, -55, -65, -79, *and*-101_. This indicates that ESBL-producing *E. coli* from Shandong province have diverse CTX-M genotypes, and similar results were also found in the tertiary and county hospitals of China (Zhang et al., 2014; Zhao et al., 2015; Wang et al., 2016).

*E. coli* isolated in this study were found to be highly resistant to penicillin derivatives, fluoroquinolone, folate pathway inhibitors, and third generation cephalosporins, but were 100% susceptible to imipenem and meropenem. In addition, these isolates displayed low resistance to amikacin, piperacillin/tazobactam, ceftazidime, and cefepime. The antibiotics to which the *E. coli* was found to be highly resistant are common medications used in Shandong town hospitals, and therefore our findings should caution clinicians for the rational use of antibiotics.

We found that *bla*_CTX-M-14_ was the most prevalent genotype of ESBL-producing *E. coli* in Shandong town hospitals, followed by *bla*_CTX-M-55_ and *bla*_CTX-M-15_, which is consistent with findings reported in Chinese county hospitals between 2010 and 2011 (Zhang et al., 2014), as well as in 3 Shanghai hospital studies between 2011 and 2013 (Zhao et al., 2015). CTX-M-55 genotype, which only has 1 amino acid site mutation (Ala-77-Val) compared to CTX-M-15 genotype, was first discovered in clinically isolated *E. coli* and *K. pneumoniae* from Thailand in 2007 (Kiratisin et al., 2007), and was subsequently detected in *Salmonella* in China, US, Korea, and Switzerland (Shi et al., 2009; Sjölund-Karlsson et al., 2011). At present, CTX-M-55 genotype is frequently detected in ESBL-producing *E. coli* that originates from animals (Dinubile et al., 2005; Ma et al., 2012; Zheng et al., 2012; Zurfluh et al., 2013; Li et al., 2016). In China, two nationwide breeding farm studies have shown that CTX-M-55 was, respectively, the second (26.1%, 29/111) and third (18.5%, 10/54) most frequently detected ESBL gene (Li et al., 2010; Zheng et al., 2012). These findings demonstrated that *bla*_CTX-M-55_ gene may have already been passed from animals to humans through the food chain. The subjects of this study were outpatients from rural town hospitals. Since residents from these regions have greater exposure to food animals and breeding farms, compared to those living in the cities, the chance of transmission of drug-resistant bacteria from animals to humans is therefore increased. However, the transmission mechanism of drug-resistant bacteria from animals to humans is currently unclear, and further studies are required to elucidate this process. Additionally, it is an interesting finding that the resistance to cefotaxime and ceftriaxone is not 100% while only CTX-M-type ESBLs were found in this study, which is needed to be further studied.

Although ST131 was the most common ST among the 201 ESBL-producing *E. coli* strains, it only accounted for 13.4% of the total ST. Similarly, some recent nationwide studies in tertiary and county hospitals have also shown that ST131 was found in 9.6% and 12.7% of ESBL-producing *E. coli*, respectively, indicating that no predominant ESBL-producing *E. coli* ST epidemic was found in China (Cao et al., 2011; Zhang et al., 2014). In contrast, the percentage of ST131 ESBL-producing *E. coli* in many European and American countries is far greater than that in China. For example, a community infection study in U.S showed that 53% of ESBL gene CTX-M-carrying *E. coli* were ST131 (Pitout et al., 2005). Another community infection study in the U.K also reported that ST131 comprised 64% of the cephalosporin-resistant *E. coli* (Lau et al., 2008b). Furthermore, a similar community infection study in Belgium between 2006 and 2007 showed that 64% of CTX-M-15-carrying *E. coli* was also ST131 (Smet et al., 2010). Of note, some sequence types found in this work belong to known international clonal complexes, such as ST131, ST393, and ST405 (Wirth et al., 2006; Hrabák et al., 2009). These international clonal complexes have been described as *E. coli* clones disseminating on a global scale (Coque et al., 2008; Nicolas-Chanoine et al., 2008; Literacka et al., 2009; Lee et al., 2010).

## Conclusions

Taken together, These findings demonstrated the high isolation rate of ESBL-producing *E. coli* (62.8%) detected in outpatients in town hospitals, China, and the *bla*_CTX-M_ gene was most dominant and was composed of a variety of subtypes. More importantly, this study spotlights the necessity to carry out long-term surveillance of ESBL-producing *E. coli* in hospital environments, especially in underdeveloped areas.

## Author contributions

ZM and SL conceived and designed the experiment. LW, YZ, and SL collected these isolates. SL, LW, YZ, and WS performed the experiments. ZM and SL analyzed the data and wrote the paper.

## Funding

This study was supported by the National Natural Science Foundations of China (81501357) and Science and Technology Development Project of Shandong Province (2014GSF118044).

### Conflict of interest statement

The authors declare that the research was conducted in the absence of any commercial or financial relationships that could be construed as a potential conflict of interest.
